# Efficacy of fentanyl transdermal patch in pain control after lower third molar surgery: A preliminary study

**DOI:** 10.4317/medoral.21161

**Published:** 2016-07-31

**Authors:** Vladimir S. Todorovic, Miroslav Vasovic, Miroslav Andric, Ljubomir Todorovic, Vladimir Kokovic

**Affiliations:** 1 Clinic of Oral Surgery, School of Dental Medicine, University of Belgrade, Serbia; 2 Department of Dentistry, Faculty of Medical Sciences, University of Kragujevac, Serbia; 3 Academy of Medical Sciences, Serbian Medical Society, Belgrade, Serbia; 4 Advanced Europe Medical Centre, Dental Department, Sharjah. College of Dental Science, Ras Al Kaimah, UAE

## Abstract

**Background:**

Surgical removal of impacted lower third molars is a common oral surgical procedure, generally followed by moderate to severe postoperative pain. Transdermal drug delivery as a concept offers interesting possibilities for postoperative pain control. The aim of this study was to evaluate the efficacy of transdermal system with fentanyl in relieving pain following impacted lower third molar surgery.

**Material and Methods:**

Seventeen patients with bilateral impacted lower third molars were included in this preliminary study. For postoperative pain control, patients randomly received a fentanyl patch plus placebo tablet after the first operation and regular (placebo) patch and an analgesic, after the second operation. Analgesia was evaluated during first 24 hours postoperatively according to patients’ reports about time of first pain appearance and additional analgesic consumption. Pain severity was rated using a 10 cm long visual analogue scale (VAS).

**Results:**

Intensity of postoperative pain and postoperative analgesic consumption were significantly lower after the Fentanyl Transdermal System (FTS) was applied (*p*<0.05). Duration of postoperative analgesia was significantly higher with FTS when compared to control treatment (*p*<0.05).

**Conclusions:**

Based on the results of this preliminary study, transdermal system with fentanyl significantly reduced postoperative pain after third molar surgery.

**Key words:**Analgesia, fentanyl, transdermal administration, third molar surgery, acute pain, postoperative care.

## Introduction

Removal of impacted lower third molars is one of the most frequently performed oral surgical procedures. Regardless the difficulty of the procedure itself, it is still so often accompanied with postoperative pain that is usually used as a model for studying analgesic properties of several drugs or physical means (1,2). Despite general progress in pain management, moderate-to-severe acute postoperative pain after removal of lower third molars remains a problem to be solved, in spite of many drugs and procedures suggested.

Transdermal drug delivery is not a new concept, but with interesting possibilities for postoperative pain control. Fentanyl was patented for use in a transdermal patch since 1984, and it has proven to be extremely effective in the treatment of severe chronic pain (3) and, interestingly, in the treatment of some postoperative pains (4,5). Fentanyl Transdermal System (FTS) is a rectangular transdermal patch containing a high concentration of fentanyl, a potent, short-acting Schedule II opiate (6). FTS includes a drug reservoir that contains Fentanyl in gel matrix, a release membrane that allows time- and surface-limited absorption of the drug, and an adhesive backing, providing a continuous systemic delivery of Fentanyl for 72 hours. It offers a prolonged and uniform analgesic effect, as well as euphoria and dysphoria (3). Patch dosage is 50 μg per hour. Because of its low molecular weight, high potency and lipid solubility, soon after application to intact skin, a Fentanyl depot concentrates in subcutaneous fat and it is then gradually released to the systemic circulation. Peek plasma concentration is reached between 24 and 72 hours of the treatment (7).

The aim of this preliminary report was to present possible efficacy of transdermal Fentanyl application for managing postoperative pain after lower third molar surgery.

## Material and Methods

The present preliminary study was carried out at the Clinic of Oral surgery, School of Dental Medicine, University of Belgrade. After study approval by the local Ethics Committee (School od Dental Medicine, University of Belgrade, No. 36/10), 17 adult patients aged between 18 and 36 years, having bilaterally impacted lower third molars indicated for surgical removal, entered the study. Following thorough information, all patients subscribed the informed consent. Ethical approval ,Ethics Committee of Faculty of Dental Medicine, University of Belgrade; No. 36/10.

Randomization was accomplished using envelope containing random number of tooth and postoperative pain control protocol. Both, the operator and patients were blinded to the use of FTS.

- Inclusion criteria

- Bilaterally identical position of impacted lower third molars;

- Good physical and mental condition (ASA I);

- Absence of infection (pericoronitis) or trauma prior to surgery.

- Exclusion criteria

- A history of allergy to the drugs used in the present study;

- A recent use of anti-inflammatory or antimicrobial drugs;

- The condition of being pregnant or lactating;

- Alcohol or narcotics abusers;

- Lack of compliance.

The lower third molars were evaluated on the panoramic radiographs in order to confirm symmetrical position on both sides. Preoperative measurements included: mouth opening dimension, taken as the maximum distance between upper and lower central incisors, and facial cheek diameter, evaluated by measuring a distance between two reference points: tragus and pogonion. All the measurements were performed by a ruler and recorded as to the nearest mm.

- Surgical procedure

In each patient, surgical extractions of bilateral impacted lower third molars were done in separate visits, the interval between surgeries being approximately two weeks. In order to avoid bias in manual skill, all surgical procedures were done by the same surgeon (first author). All the procedures were performed in local anesthesia using 2% lidocaine chloride with adrenaline 1:80.000 (Lidokain-adrenalin®, Galenika a.d., Belgrade, Serbia), without any premedication or use of sedation during surgery. After raising a mucoperiosteal flap from the buccal aspect, a round bur with sterile saline irrigation was used to remove bone over the impacted tooth. If needed, sectioning of the crown and roots was performed before removing the tooth. The extraction wound was inspected and irrigated with sterile saline solution before suturing with a 4-0 suture. All the data concerning surgery were recorded, including the duration of surgery from the incision to placement of the last interrupted suture.

- Postoperative regimen and measurements

Postoperative pain control regimens were randomly divided into two groups (FTS and control group). After the first operation, as the FTS group, all the patients received a transdermal patch containing Fentanyl (FTS - Durogesic®, Janssen Pharmaceutics, Belgium) and a placebo tablet postoperatively. After the second operation, patients received a regular (placebo) patch and an analgesic tablet - a non-steroid anti-inflammatory drug (Diclofenac Duo® 75mg, Pharmaswiss, Czech Republic), orally once daily, representing a control regimen. Patients were advised to take an additional analgesic tablet as soon as their pain reached a moderate level, recording the exact moment of taking additional analgesic, so that we could count the elapsed time till the occurrence of pain. After each operation, patients were instructed to record the number of tablets used.

Concerning antimicrobial therapy, all the patients were given amoxicillin 500 mg every 8 h orally for 5 days and advised to use chlorhexidine mouth rinse twice daily starting on the day after each operation for 10 following days. The patients were given usual postoperative instructions.

Each patient was evaluated at the follow-up control 24 hours postoperatively. Postoperative pain was evaluated using a 100 mm long visual analogue scale (VAS) with verbal descriptors “no pain” at the left end of the scale and “extremely severe pain” at the right end. The same examiner who assessed the patients preoperatively performed clinical measurements during follow-up examinations. Trismus and facial edema, measured as stated previously, were recorded as the differences between preoperative (baseline) and postoperative (after 24 hours) values.

- Statistical analysis

Statistical analysis was performed using the Statistical Package for Social Sciences (SPSS for Windows, version 20.0; SPSS Inc., Chicago, IL, USA). The normality of the data was evaluated using Shapiro–Wilk test. Differences between two protocols were analyzed by Wilcoxon signed rank test for paired samples and the paired t-test, depending on data normality. Categorical variables were analyzed by χ2 test. Statistical differences between groups were accepted for *p*-values <0.05.

## Results

Data from 17 patients (12 females, 5 males) were included in the study, the mean age being 22.8 ± 4.2 years. There was no statistically significant difference between study groups with regard to duration of surgery in both sessions ( Table 1). A postoperative course was uneventful in all patients. However, when received a transdermal patch containing Fentanyl (FTS), patients experienced postoperative pain much more rarely than after surgery when received only a non-steroid anti-inflammatory drug.

**Table 1:**
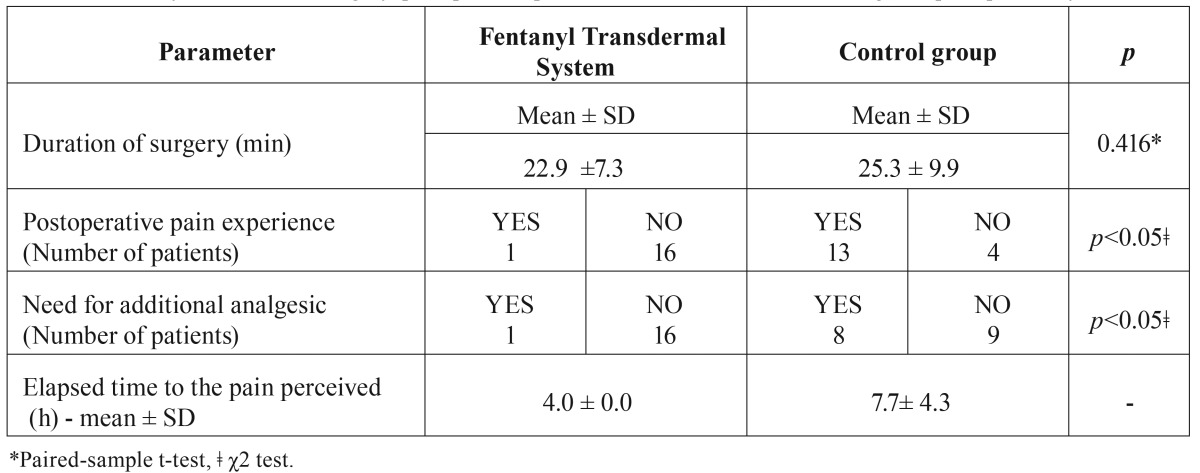
Summary of duration of surgery, postoperative pain and the need for additional analgesics postoperatively.

With respect to pain evaluation by the VAS, there was a statistically significant difference between the FTS and the control group after 24 hours ( Table 2). However, there was no statistically significant difference regarding postoperative facial swelling and trismus between the groups.

**Table 2:**
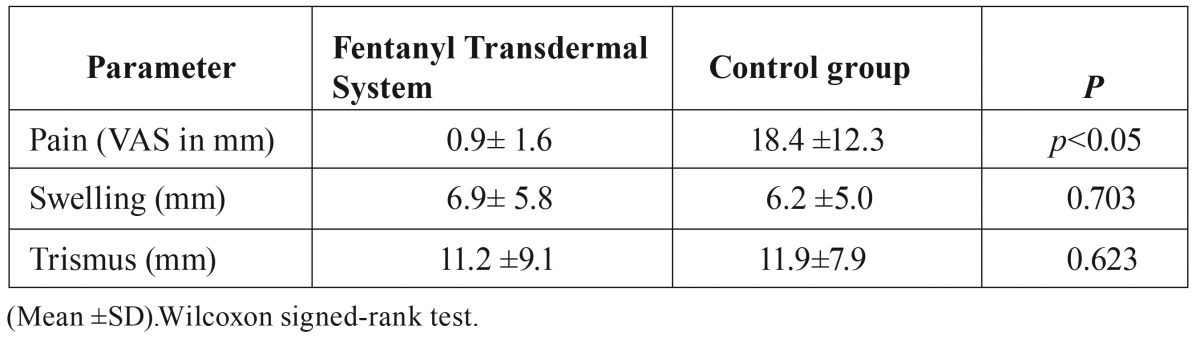
Pain, swelling and trismus 24 hours after each surgery in the investigated patients.

## Discussion

The impact that postoperative pain, among other postoperative sequels, has on patient quality of life after lower third molar surgery is undoubted. Of all the symptoms associated with this procedure, postoperative pain is one that patients apprise as the most inconvenient. In the literature, there are many articles regarding control of postoperative pain using various analgesics over time and comparing their efficacy. Theoretically, FTS could be an ideal drug for patients who are unable to eat due to severe trismus, swelling and especially for patients with gastrointestinal problems such as ulcer.

Although the efficacy of FTS has already been proven in the treatment of chronic cancer pain (8-10), there have been relatively few reports evaluating the effectiveness of FTS for the treatment of acute postoperative pain. According to the best of our knowledge, there is not any report in the literature on use of FTS for pain control after lower third molar surgery. However, there are few considerations that have to be discussed.

Adverse events due to transdermal fentanyl use can be divided into three categories based largely on the intent of use: appropriate therapeutic use, inappropriate therapeutic use (misuse), and abuse. Abuse is defined as the intentional inappropriate use of the transdermal device, or its contents, for purposes other than those for which the transdermal device was intended or prescribed (11). This is typically done with euphoric intent, though it may occasionally be for suicidal reasons. Besides euphoric intent, other side effects such as skin irritation, nausea, fever, headache and clinically relevant respiratory depression are also described in the literature (11). Case reports detail that elevation in skin or ambient temperatures from external sources, such as hot tubs or heating blankets, may lead to fentanyl overdose (12). In our study, patients were distinctly informed about the procedure and possible side effects, thus monitoring of each patient was organized in agreement with family member. In very few cases, nausea was reported, especially when patients were moving, which was stopped at moment of siting. Other side effects were not reported.

The recipient site on the skin that is most appropriate for application of FTS and its condition should also been discussed. The average skin thickness of the human body is 40 μm, but it ranges between 20 and 80 μm based on location, race, age, and gender, among other factors. In skin samples from 8 individuals, there was a >50% difference in the permeability of fentanyl (13). Skin surface areas with similar stratum corneum thickness typically possess similar diffusion rates within an individual, explaining why the chest, extremities, and abdomen are acceptable sites for transdermal device application without the need for any dosage changes (13,14).

Also, following application of a transdermal fentanyl device to broken skin, blood fentanyl concentrations can rise 5-fold (15). In our study, as a recipient site, skin of the upper arm was used, after meticulous inspection of the area.

Recently, Fentanyl Iontophoretic Transdermal System (fentanyl ITS) was introduced as a system that has been approved especially for the management of acute, moderate-to-severe postoperative pain (16). Fentanyl ITS is the first needle-free, self-contained, patient-activated system that delivers fentanyl directly through the skin by application of a low-intensity electrical field (17). Future investigations should consider the use of Fentanyl ITS, as it allows patients to maintain an acceptable level of pain control following titration to comfort with a loading dose of opioid, while avoiding the first-pass effect and analgesic gaps associated with a delayed passive delivery using FTS patch.

In conclusion, it can be stated that this preliminary report found FTS to be very effective in relieving postoperative pain after lower third molar surgery, with excellent tolerability. However, future clinical trials are necessary, with sufficient sample size and use of fentanyl ITS, regarding possible standard use of opioids for control of acute pain after oral surgery.
